# Supplementary material for: Histone Deacetylase Inhibitors as a Therapeutic Strategy to Eliminate Neoplastic “Stromal” Cells from Giant Cell Tumors of Bone
Source: Cancers (Basel). 2022 Sep 27;14(19):4708. doi: 10.3390/cancers14194708 (PMC9563449; doi:10.3390/cancers14194708)
Supplement: Supplementary file 1 [file cancers-14-04708-s001.zip › cancers-1924096-Supplementary Materials - Figures_Proofread SV.pdf]

Supplementary Materials - Figures

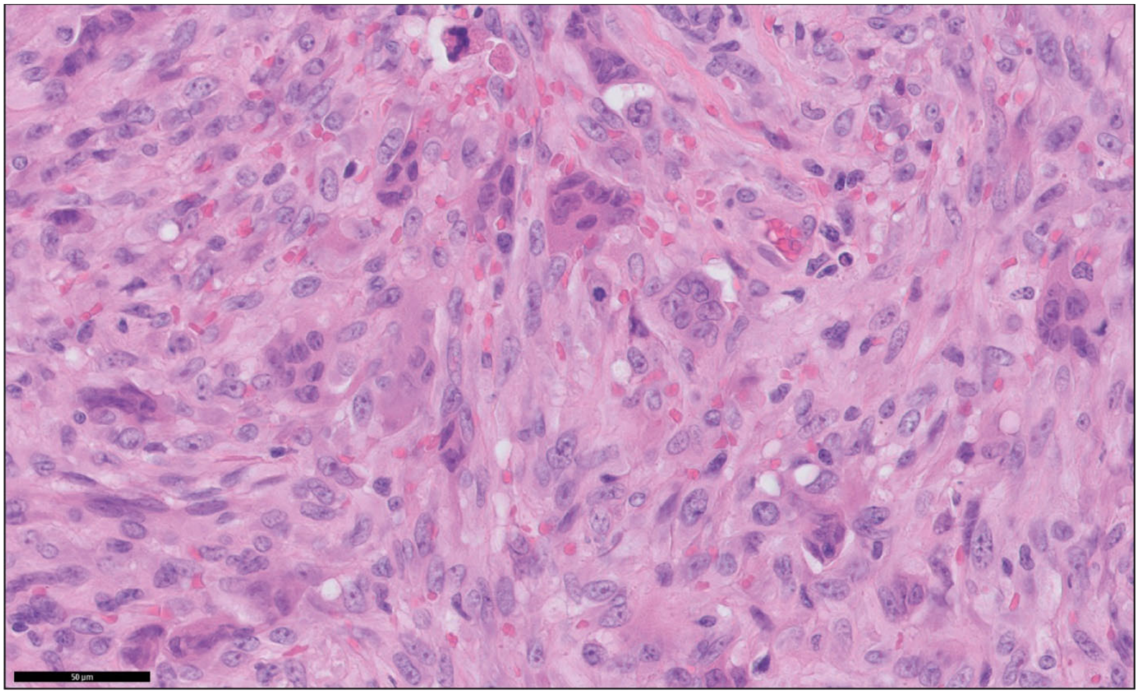

Figure S1. Original tumor of L4040 was diagnosed as GCTB with atypia.
Histology showed areas with epithelioid and spindled cells with large nuclei with coarse, open chromatin, displaying increased mitotic activity, admixed with multinucleated giant cells. Since significant nuclear atypia, hyperchromasia, atypical mitoses and infiltration of preexisting bone were absent, the morphology was interpreted as GCTB with atypia, not fulfilling the criteria of malignant GCTB. Scale bar: 50 μm.

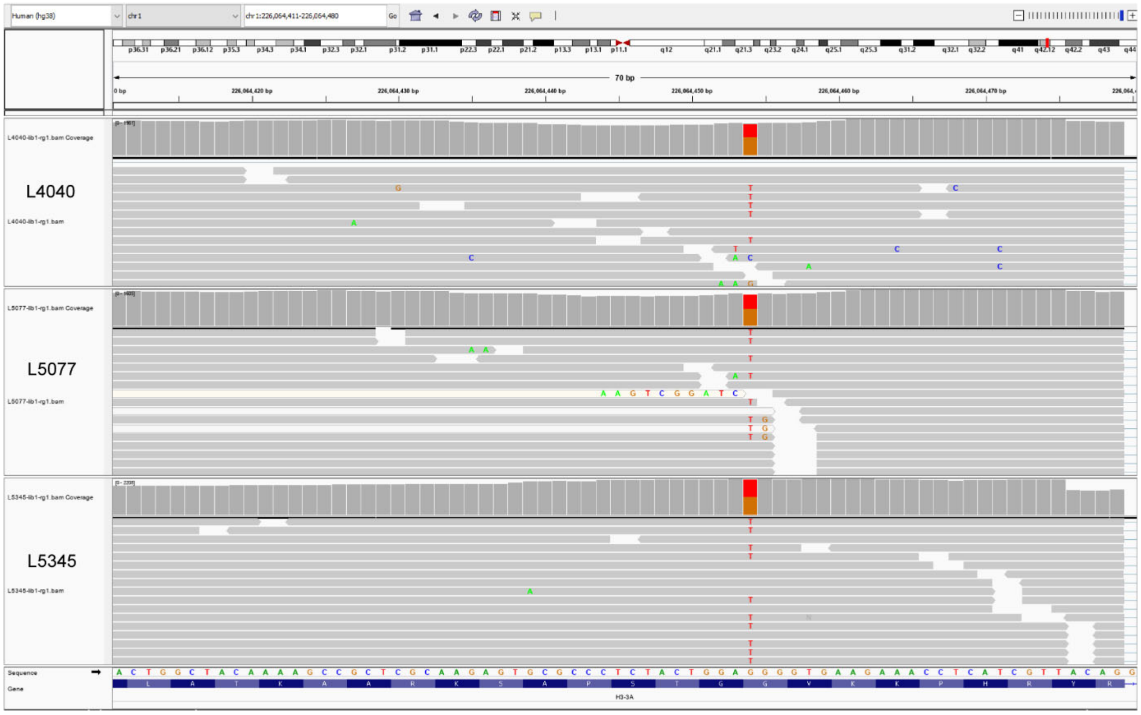

Figure S2. Established cell lines show RNA expression of H3F3A-WT and H3F3A p.G34W. Transcriptome reads of H3F3A c.103G>T mutation resulting in H3F3A p.G34W in L4040 (p100), L5077 (p28), and L5345 (p27) cells. Data were extracted from a previously generated RNA-sequencing dataset. L5345 cells have a balanced expression of wildtype and mutant transcripts (51% vs. 49%, respectively).

A **Whole Blots Figure 4B**

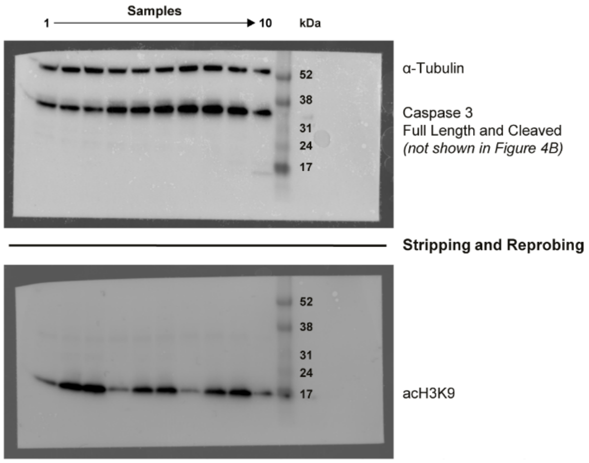

			α-Tubulin	acH3K9	
		Treatment	Intensity	Intensity	Ratio
1	L4040	-	3710377	9112600	2.46
2		3 nM romidepsin	5329740	25731450	4.83
3		30 nM panobinostat	5624658	27125112	4.82
4	L5862	-	4591180	8298822	1.81
5		1 nM romidepsin	3867880	17437013	4.51
6		50 nM panobinostat	4526332	19121718	4.22
7	L5077	-	6452472	6524524	1.01
8		1 nM romidepsin	6104818	19087695	3.13
9		20 nM panobinostat	4434417	23114112	5.21
10	Neg. Ctrl		3336364	8715300	2.61

B **Whole Blots Figure 4C**

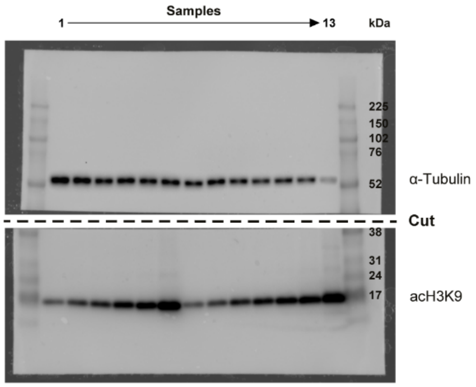

			α-Tubulin	acH3K9	
		Romidepsin (nM)	Intensity	Intensity	Ratio
1	L4040	-	5367720	5636065	1.05
2		0.32	4758720	8785767	1.85
3		0.56	3530732	10895018	3.09
4		0.75	4050759	15952362	3.94
5		1	3609177	18775926	5.20
6		3.2	3897173	29152517	7.48
7	L5862	-	3018461	7318295	2.42
8		0.1	3495086	8620455	2.47
9		0.32	2780825	12170563	4.38
10		0.56	2351880	14432640	6.14
11		0.75	2521338	17769031	7.05
12		1	2064391	20528290	9.94
13	Pos. Ctrl		596222	25899577	43.44

C **Whole Blots Figure 4D**

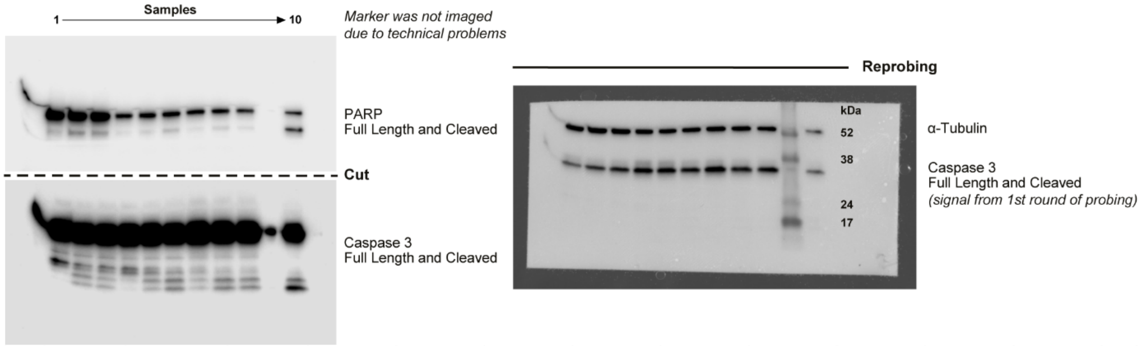

			α-Tubulin	PARP (full)		PARP (cleaved)		Caspase 3 (full)		Caspase 3 (cleaved)	
		Treatment	Intensity	Intensity	Ratio	Intensity	Ratio	Intensity	Ratio	Intensity	Ratio
1	L4040	-	8505160	13261974	1.56	621951	0.073	14568592	1.71	78030	0.009
2		3 nM romidepsin	10174386	12634116	1.24	1280400	0.126	13052668	1.28	583083	0.057
3		30 nM panobinostat	9517442	12730971	1.34	1070025	0.112	13651068	1.43	354165	0.037
4	L5862	-	8087578	3812358	0.47	224169	0.028	15267122	1.89	98634	0.012
5		1 nM romidepsin	7744172	4235814	0.55	264165	0.034	15987820	2.06	586534	0.076
6		50 nM panobinostat	8247254	4421340	0.54	498003	0.060	14262014	1.73	1186109	0.144
7	L5077	-	9395994	3693459	0.39	140745	0.015	20037424	2.13	348536	0.037
8		1 nM romidepsin	8840168	3367452	0.38	229845	0.026	18457043	2.09	1269238	0.144
9		20 nM panobinostat	7742956	2383029	0.31	107316	0.014	17409564	2.25	1060960	0.137
13	Pos. Ctrl		3188124	1730322	0.54	1713822	0.538	8907592	2.79	2486074	0.780

Figure S3. Original blots from all performed Western blots.
Whole blots with densitometry readings from the western blots depicted in (A) Figure 4B, (B) Figure 4C, and (C) Figure 4D.
