# Supplementary material for: Histone Deacetylase Inhibitors as a Therapeutic Strategy to Eliminate Neoplastic “Stromal” Cells from Giant Cell Tumors of Bone
Source: Cancers (Basel). 2022 Sep 27;14(19):4708. doi: 10.3390/cancers14194708 (PMC9563449; doi:10.3390/cancers14194708)
Supplement: Supplementary file 1 [file cancers-14-04708-s001.zip › cancers-1924096-Supplementary Materials - Tables_Proofread SV.pdf]

Supplementary Materials - Tables

Table S1. Clinical information of patient samples that were used to establish cell lines of neoplastic “stromal” cells.

Sample ID	Gender	Age at Diagnosis	Tumor Type	Tumor Location	Primary or Recurrence?	Pre-Operative Treatment	H3F3A Mutation	Doubling Time of Cell Line
L4040	Female	42	GCTB with atypia*	Ulna	Recurrence	Denosumab	p.G34W	1.4 days
L5077	Female	25	GCTB	Tibia	Primary	Denosumab	p.G34W	4.2 days
L5345	Male	26	GCTB	Femur	Primary	Denosumab	p.G34W	4.9 days
L5862	Female	17	GCTB	Vertebra	Primary	Denosumab	p.G34W	1.8 days

*See Figure S1 for histology.

Table S2. STR profiles of corresponding primary tumor tissues and established cell lines.

Sample ID	Specimen Type	Passage Number	Amelogenin	CSF1PO	D13S317	D16S539	D21S11	D5S818	D7S820	TH01	TPOX	vWA
L4040	Primary	-	X	9,12	11	11,12	28,30	11,13	8,10	6,9.3	8,11	15
	Cell line	P109	X	9,12	11	11,12	28,30	11,13	8,10	6,9.3	8,11	15
L5077	Primary	-	X	11,12	11,12	11,12	29	12,13	8,11	6,7	8	16
	Cell line	P36	X	11,12	11,12	11,12	29	12,13	8,11	6,7	8	16
L5345	Primary	-	X,Y	11	11,13	11	29,34.2	11,12	8,11	9	11	17
	Cell line	P27	X,Y	11	11,13	11	29,34.2	11,12	8,11	9	11	17
L5862	Primary	-	X	11,12	11	11,12	30,31	9,11	11,12	9,9.3	8	17,18
	Cell line	P10	X	11,12	11	11,12	30,31	9,11	11,12	9,9.3	8	17,18

Table S3. Detailed list of all compounds included in the epigenetics compound library (L1900, Selleckchem).

		Product Name	Specific Targets
**Aurora Kinases**	1	Alisertib	Aurora A
	2	Aurora A Inhibitor I	Aurora A
	3	MK-5108	Aurora A
	4	Danusertib	Aurora A, Abl, RET, TrkA, FGFR1, Aurora C, Aurora B
	5	MLN8054	Aurora A, Aurora B
	6	CCT129202	Aurora A, Aurora B, Aurora C
	7	ZM 447439	Aurora A, Aurora B, LCK, Src, MEK1
	8	CYC116	Aurora A, Aurora B, VEGFR2, FLT3, CDK2, CDK9, p70 S6K
	9	CCT137690	Aurora A, Aurora C, Aurora B
	10	PHA-680632	Aurora A, Aurora C, Aurora B, FGFR1, PLK1, FLT3, VEGFR3, VEGFR2, LCK
	11	Tozasertib	Aurora A, Aurora C, Aurora B, FLT3, Bcr-Abl
	12	Barasertib	Aurora B
	13	Hesperadin	Aurora B
	14	SNS-314 Mesylate	Aurora C, Aurora A, Aurora B
	15	AMG-900	Aurora C, Aurora B, Aurora A, p38α
**Bromodomain and Extra-Terminal Motif Proteins**	16	RVX-208	BD2
	17	I-BET-762	BRD2, BRD3, BRD4
	18	OTX015	BRD2, BRD3, BRD4
	19	PFI-1	BRD2, BRD4
	20	I-BET151	BRD3, BRD2, BRD4
	21	(+)-JQ1	BRD4
	22	CPI-203	BRD4, IL-6, MYC
	23	Bromosporine	CECR2, BRD9, BRD4, BRD2
**Catechol-O-methyltransferases**	24	Entacapone	Catechol-O-methyltransferase (COMT)
**Cyclin-dependent Kinases**	25	JNJ-7706621	CDK2, CDK1, Aurora A, Aurora B, CDK3, VEGFR2, CDK6, FGFR2, CDK4, GSK-3β, Tie-2, FGFR1, VEGFR3
**DNA Methyltransferases**	26	Azacitidine	DNA Methyltransferase
	27	Decitabine	DNA Methyltransferase
	28	RG108	DNA methyltransferase
	29	Zebularine	DNA Methyltransferase, Cytidine deaminase
	30	Procainamide HCl	DNA methyltransferase, Sodium channel
	31	SGI-1027	DNMT1, DNMT3B, DNMT3A
**Epidermal Growth Factor Receptor**	32	AG-490	EGFR
	33	CUDC-101	EGFR, HDAC1, HDAC6, HDAC3, HDAC5, HDAC2, HDAC4, HER2, HDAC10, HDAC9, HDAC8, HDAC7
	34	WHI-P154	EGFR, VEGFR, Src, JAK3
**Fms Related Receptor Tyrosine Kinase 3**	35	KW-2449	FLT3, Abl, FGFR1, Aurora A, JAK2, Kit, Src
	36	Pacritinib	FLT3, JAK2, TYK2, JAK3
	37	ENMD-2076	FLT3, RET, Aurora A, VEGFR3, Src, NTRK1, CSF-1R, LCK, FAK, PDGFRα, VEGFR2, BLK, FGFR2, YES1, Abl1, FGFR1, Fyn, JAK2, Kit, Aurora B
**Histone Acetyltransferases**	38	SGC-CBP30	CREBBP, EP300
	39	C646	p300/CBP
**Histone Deacetylases**	40	AR-42	HDAC
	41	Belinostat	HDAC

	42	Dacinostat	HDAC
	43	M344	HDAC
	44	Panobinostat	HDAC
	45	Scriptaid	HDAC
	46	Sodium Phenylbutyrate	HDAC
	47	Vorinostat	HDAC
	48	Givinostat	HDAC (Class I, IIA, IIB)
	49	Trichostatin A	HDAC (Class I, IIA, IIB)
	50	MC1568	HDAC (Class IIA)
	51	Valproic Acid	HDAC, Autophagy, GABA Receptor
	52	Romidepsin	HDAC1, HDAC2
	53	Quisinostat	HDAC1, HDAC2, HDAC11, HDAC10, HDAC4, HDAC5, HDAC8, HDAC3
	54	Mocetinostat	HDAC1, HDAC2, HDAC11, HDAC3
	55	Tacedinaline	HDAC1, HDAC2, HDAC3
	56	Entinostat	HDAC1, HDAC3
	57	Fimepinostat	HDAC1, HDAC3, HDAC10, HDAC2, HDAC11, PI3Kα, HDAC6, PI3Kδ, PI3Kβ
	58	Resminostat	HDAC1, HDAC3, HDAC6
	59	Abexinostat	HDAC1, HDAC3, HDAC6, HDAC2, HDAC10, HDAC8
	60	Pracinostat	HDAC10, HDAC3, HDAC5, HDAC1, HDAC4, HDAC9, HDAC11, HDAC2, HDAC7, HDAC8
	61	RGFP966	HDAC3
	62	RG2833	HDAC3, HDAC1
	63	Nexturastat A	HDAC6
	64	Rocilinostat	HDAC6
	65	Tubacin	HDAC6
	66	Tubastatin A	HDAC6
	67	Tubastatin A HCl	HDAC6
	68	PCI-34051	HDAC8
	69	Droxinostat	HDAC8, HDAC6, HDAC3
	70	TMP269	HDAC9, HDAC7, HDAC5, HDAC4
**Histone Demethylases**	71	OG-L002	KDM1A
	72	IOX1	KDM3A, KDM4C, KDM6B, KDM2A, KDM4E, KDM5C, PHD2
	73	GSK J4 HCl	KDM6B
**Histone Methyltransferases**	74	BIX 01294	KMT1C
	75	MM-102	KMT2A
	76	EPZ004777	KMT4
	77	EPZ5676	KMT4
	78	SGC 0946	KMT4
	79	EPZ-6438	KMT6
	80	3-Deazaneplanocin A	KMT6, S-adenosylhomocysteine hydrolase
**Hypoxia-inducible Factors**	81	IOX2	HIF-1α prolyl hydroxylase-2
	82	2-Methoxyestradiol	HIF-2α, Microtubules depolymerisation, HIF-1α
	83	FG-4592	HIF-α prolyl hydroxylase
**Janus Kinases**	84	CYT387	JAK1, JAK2, JAK3
	85	Filgotinib	JAK1, JAK2, TYK2, JAK3
	86	AZ 960	JAK2
	87	AZD1480	JAK2
	88	CEP-33779	JAK2
	89	LY2784544	JAK2, FLT3, JAK1, FLT4, FGFR2, TYK2, JAK3, TrkB, FGFR3, KDR, ALK, MUSK, Aurora A, MAP3K9
	90	TG101348	JAK2, FLT3, RET

	91	Ruxolitinib	JAK2, JAK1
	92	S-Ruxolitinib	JAK2, JAK1, TYK2
	93	Baricitinib	JAK2, JAK1, TYK2, JAK3
	94	XL019	JAK2, PDGFRβ, JAK1, FLT3, JAK3
	95	TG101209	JAK2, RET, FLT3, JAK3
	96	WP1066	JAK2, STAT3
	97	NVP-BSK805 2HCl	JAK2, TYK2, JAK3, JAK1
	98	TAK-901	JAK3, c-Src, CLK2, FGR, YES1, LRRK2, FLT3, Fyn, ARG, Axl, Hck, SNF1LK2, Abl, RET, TrkA, LCK, PTK5, Fms, FGFR2, EphB1, EphA1, ARK5, ITK, ALK2, CDK7, BLK, Aurora B, JAK2, EphB2, Aurora A, STK16, EphA2, BRK, EphB4, TNK2, FGFR1, EphA4, STK33, CLK1, AMPK, FES, SLK, Chk2, TYK2, BTK, FAK2, c-Kit, VEGFR2, FGFR3, ALK5, MAP3K9, CLK3, JAK1
	99	AT9283	JAK3, JAK2, Aurora A, Aurora B, Abl1, GSK-3β, FGFR2, VEGFR3, Mer, RET, RSK2, RSK3, TYK2, YES, Abl, DRAK1, FGFR1, FGFR2, FGFR3, VEGFR1, FLT3, PDGFRα, PDK-1, PKCμ, RSK4, Src, VEGFR2
	100	Tofacitinib	JAK3, JAK2, JAK1
	101	Tofacitinib Citrate	JAK3, JAK2, JAK1
	102	ZM 39923 HCl	TGM2, JAK3, EGFR, JAK1
**Lethal(3)Malignant Brain Tumor-Like Proteins**	103	UNC669	L3MBTL1, L3MBTL3, L3MBTL4
	104	UNC1215	L3MBTL3
**Monoamine Oxidases**	105	Tranylcypromine HCl	MAO-B, MAO-A, LSD1
**O6-Alkylguanine DNA Alkyltransferases**	106	Lomeguatrib	MGMT
**Poly(ADP-Ribose) Polymerases**	107	3-Aminobenzamide	PARP
	108	AZD2461	PARP
	109	INO-1001	PARP
	110	PJ34	PARP
	111	PJ34 HCl	PARP
	112	Rucaparib	PARP
	113	AG-14361	PARP1
	114	Iniparib	PARP1
	115	BMN 673	PARP1, PARP2
	116	Olaparib	PARP2, PARP1
	117	UPF 1069	PARP2, PARP1
	118	Veliparib	PARP2, PARP1
	119	ME0328	PARP3, PARP1
**Pim Kinases**	120	SMI-4a	Pim1
	121	SGI-1776 free base	Pim1, FLT3, Pim3, Pim2
	122	AZD1208	Pim1, Pim3, Pim2
	123	CX-6258 HCl	Pim1, Pim3, Pim2
**Sirtuins**	124	Selisistat	SIRT1
	125	SRT1720	SIRT1
	126	Resveratrol	SIRT1, SIRT2, Quinonen reductase 2, IKK β, COX1, COX2, DNA poymerase α, LOX
	127	Quercetin	SIRT1, Src, PKC, PI3Kγ, PI3Kδ, PI3Kβ
	128	Sirtinol	SIRT2, SIRT1

The specific targets of the compounds are listed in order of increasing IC50 values determined in cell-free kinase activity assays (Selleckchem website).
